# Head and neck cancer in the UK: what was the stage before COVID-19? UK cancer registries analysis (2011-2018)

**DOI:** 10.1038/s41415-022-5151-4

**Published:** 2022-11-11

**Authors:** Grant Creaney, Alex D. McMahon, Alastair J. Ross, Lesley A. Bhatti, Claire Paterson, David I. Conway

**Affiliations:** 4141575499001 Clinical Lecturer in Dental Public Health, School of Medicine, Dentistry and Nursing, University of Glasgow, Glasgow, UK; 4141575499002 Reader (Dental School), School of Medicine, Dentistry and Nursing, University of Glasgow, Glasgow, UK; 4141575499003 Senior Lecturer in Human Factors in Health Care, School of Medicine, Dentistry and Nursing, University of Glasgow, Glasgow, UK; 4141575499004 Senior Statistician, Public Health Scotland, Edinburgh, UK; 4141575499005 Consultant Clinical Oncologist, Beatson West of Scotland Cancer Centre, NHS Greater Glasgow and Clyde, UK; 4141575499006 Professor of Dental Public Health,, School of Medicine, Dentistry and Nursing, University of Glasgow, Glasgow, UK

## Abstract

**Introduction**People who present with more advanced stage head and neck cancer (HNC) are associated with poorer outcomes and survival. The burden and trends of advanced stage HNC are not fully known at the population level. The UK national cancer registries routinely collect data on HNC diagnoses.

**Aims**To describe trends in stage of diagnosis of HNCs across the UK before the COVID-19 pandemic.

**Methods ** Aggregated HNC incidence data were requested from the national cancer registries of the four UK countries for the ten most recent years of available data by subsite and American Joint Commission on Cancer stage at diagnosis classification. Additionally, data for Scotland were available by age group, sex and area-based socioeconomic deprivation category.

**Results ** Across the UK, rates of advanced stage HNC had increased, with 59% of patients having advanced disease at diagnosis from 2016-2018. England had a lower proportion of advanced disease (58%) than Scotland, Wales or Northern Ireland (65-69%) where stage data were available. The completeness of stage data had improved over recent years (87% by 2018).

**Conclusion**Prior to the COVID-19 pandemic, diagnoses of HNC at an advanced stage comprised the majority of HNCs in the UK, representing the major challenge for the cancer healthcare system.

**Supplementary Information:**

Zusatzmaterial online: Zu diesem Beitrag sind unter 10.1038/s41415-022-5151-4 für autorisierte Leser zusätzliche Dateien abrufbar.

## Key points

- From 2009-2018, 59% of patients with head and neck cancer in the UK were diagnosed with advanced stage disease where the stage was recorded in national cancer registries.
- The proportion of cancers diagnosed with advanced disease in 2016-2018 was highest in Wales (69%), followed by Northern Ireland (67%), Scotland (65%) and then England (58%), where the stage was recorded.
- Patients with oral cavity cancer or oropharyngeal cancers were more likely to have advanced disease than early disease.
- Data on head and neck cancer stages in UK cancer registries have improved in completeness in recent years, with 87% known by 2018.

**Supplementary Information:**

Zusatzmaterial online: Zu diesem Beitrag sind unter 10.1038/s41415-022-5151-4 für autorisierte Leser zusätzliche Dateien abrufbar.

## Introduction

Head and neck cancer (HNC) - the collective terms for cancers of the oral cavity, oropharynx, larynx and other sites of the head and neck - is the seventh most common cancer globally, with incidence rates staying either static or rising over recent decades and being the ninth highest cause of cancer-related mortality.^1^ In the UK, it is estimated that approximately 12,200 people have a new diagnosis of HNC every year, a 33% rise in incidence since the early 1990s,^2^ which seems to be largely driven by dramatic increases in oropharyngeal cancer. The five-year survival for patients with HNC, although improving, remains poor globally,^3^ with only a 28-67% chance of survival at five years in the UK depending on the subsite^4^ and mortality rates in Scotland rising by 12% in women and 22% in men over the last decade. Many patient, tumour and treatment factors contribute towards survival outcomes with HNC, with a key prognostic tumour indicator being the stage of disease at diagnosis.^5^

Early-stage HNC, where there is no locoregional spread, is associated with relatively more straightforward treatment and better prognosis. Conversely, advanced-stage HNC is associated with more complex/involved treatment and management, or in some cases, palliative care.^6^ Advanced-stage disease also substantially impacts on quality of life and poorer survival outcomes.^7^

Globally, a number of studies have shown high proportions of HNC diagnosed at advanced stage has remained high, including a large cohort from South America (2011-2017) with 75% advanced stage,^8^and a multicentre European case-control study (2002-2004) with 54%.^9^In the UK, two historic large clinical cohorts - one in Scotland 1999-2000^10^ and one in South West England 1996-2003^11^ - reported 56% and 56-59% advanced-stage HNC, respectively. More recently, the UK-wide Head and Neck 5000 study (HN5000) cohort study 2011-2014 recruited 59.6% people with advanced-stage disease.^12^ However, thus far, population cancer registry data have not been utilised to examine stages of HNC in the UK.

Opportunistic oral soft tissue screening is undertaken by dentists when examining patients and is important in detecting early-stage tumours;^13^ however, it is appreciated that the rarity of these cancers and high number of sufferers who visit dental/health practitioners infrequently before diagnosis means that more targeted measures of early diagnosis may be more appropriate.^14^ HNC can be cured more readily if the tumour is diagnosed at an early stage and confined to the head and neck region but survival is poor if there is late-stage disease, metastatic spread and recurrence of disease.^15^

We aimed to describe the stage at diagnosis of HNCs in the UK at the population level before the COVID-19 pandemic by subsite, age, sex, socioeconomic factors and over time. We also aimed to assess the completeness and availability of HNC stage data held by the cancer registries of the UK.

## Methods

Information requests were submitted to the Scottish Cancer Registry (Public Health Scotland),^16^ English Cancer Registry National HNC report (Public Health England),^17^ Welsh Cancer Intelligence and Surveillance Unit (Public Health Wales)^18^ and the Northern Ireland Cancer Registry (Queen's University, Belfast).^19^ Data were requested where available on the numbers of cases and both the crude and European age-standardised incidence rates (per 100,000 population) of HNC over the most recent ten-year period (2009-2018), by HNC subsite and stage of diagnosis. Subsites were defined via the International Classification of Diseases (ICD)^20^ as: oral cavity cancers (C00.3, C00.4, C02.0, C02.1, C02.2, C03.0, C03.1, C04.0, C04.1, C05.0, C06.0, C06.1, C06.2); oropharyngeal cancer (C01, C02.4, C05.1, C05.2, C09.0, C09.1, C09.9, C10.0, C10.1, C10.2, C10.3); laryngeal cancer (C32.0, C32.1, C32.2); and for other sites of the head and neck (C07, C08.0, C08.1, C08.8, C08.9, C11.0, C11.1, C11.2, C11.3, C12, C13.0, C13.1, C13.2, C14.0, C14.8, C30.0, C31.0, C31.1, C73X).

HNC is staged using the American Joint Commission on Cancer's TNM Classification of Malignant Tumours (TNM)^7^^,^^21^ in order to categorise an individual's disease into one of four stages, from stage I to stage IV at the time of diagnosis. TNM is used in treatment planning and gives a clinical description of the disease by tumour size, whether there is nodal involvement, and whether the cancer has metastasised to another site in the body. Stage I and II HNCs are considered early stage disease and are associated with more straightforward treatment and better prognosis, while stage III and IV HNCs are late-, or more accurately termed, advanced-stage disease.

The Scottish Cancer Registry HNC data were also available by the additional demographics of age (five-year age bands), sex (male, female) and area-based socioeconomic measure - Scottish Index of Multiple Deprivation (SIMD) fifths - where SIMD-1 is the most deprived and SIMD-5 is the least deprived.^22^

Crude incidence rates of HNCs were collated by stage for 2009-2018 where data were available. Age-standardised rates (ASR) were available for England, Scotland and Northern Ireland. Where data were incomplete, this was factored into analysis in the description. ASR provide the incidence rate of a disease per 100,000 person-years within a population standardised to a standard population (European ASR [EASR] being standardised to a European population), which allows comparison between different regions and countries which might have a different underlying population age-structure.

Further aggregated data were available for Scotland from years 2016-2018 covering age, sex, stage at diagnosis and SIMD quintile. Odds ratios (OR) and 95% confidence intervals (95% CI) were calculated for this dataset to ascertain any inequalities that could exist. Only cases where the stage was known were included for analysis.

Least square means were calculated for ASR data from England, Northern Ireland and Scotland using SAS 16.

## Results

Incidence by crude count was available for all four nations for all HNCs; however, data for Wales were only available for a slightly different International Classification of Diseases specification (C00-C14, C30-32). Incidence by subsite was available for England, Northern Ireland and Scotland. Incidence by EASR was available for stages of diagnosis from England, Northern Ireland and Scotland. Scotland had no data on stages available before 2016 (Table 1). Staging data were incomplete for all countries. For staging collations and comparisons, data from 2016-2018 has been used. All data utilised for stage analyses are shown in online Supplementary Table 1.

**Table 1:** HNC stage-related data available for analysis from information requests

**Country**	**Counts**	**Crude rate**	**EASR**	**Years available**	**Subsite**	**Stage data routinely published**	**Socioeconomic status**	**Age**	**Sex**
England	Yes	Yes	Yes	2009-2018	Yes	Partial	No	No	No
Northern Ireland	Yes	Yes	Yes	2009-2018	Yes	Partial	No	No	No
Scotland	Yes	Yes	Yes	2016-2018	Yes	No	Yes^*^	Yes^*^	Yes^*^
Wales	Yes	No	No	2011-2018	Yes	Partial	No	No	No
Key: * = Specifically requested									

**Fig. 1:**
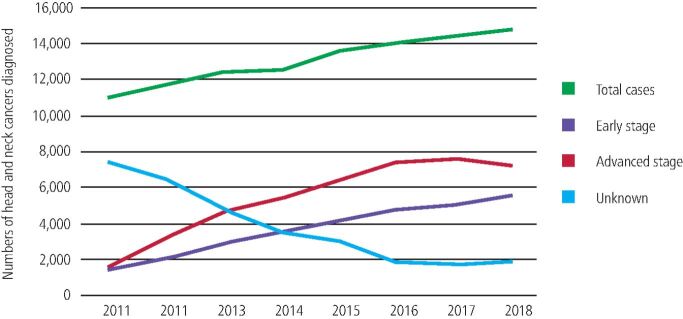
Numbers of new HNCs in the UK from 2011-2018 by stage at diagnosis

A total of 104,913 cases of HNC were diagnosed across England, Scotland, Wales and Northern Ireland from 2011-2018. Diagnosis across all subsites was more common to be at an advanced stage rather than early stage, apart from for laryngeal cancers. Across all nations and subsites together, 59.0% of new HNCs were diagnosed at an advanced stage where the stage was known from 2016-2018 (Fig. 1).

**Fig. 2:**
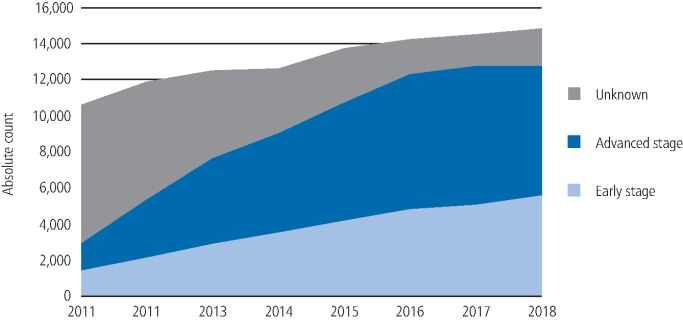
Proportion of HNCs in the UK from 2011-2018 by stage at diagnosis

In 2011, only 32.6% of new HNC cases recorded in cancer registries had a stage at diagnosis recorded. This improved to 86.9% by 2018. As stage data became more complete over the seven years, rates of advanced disease rose much more quickly than early stages (Fig. 2).

**Fig. 3:**
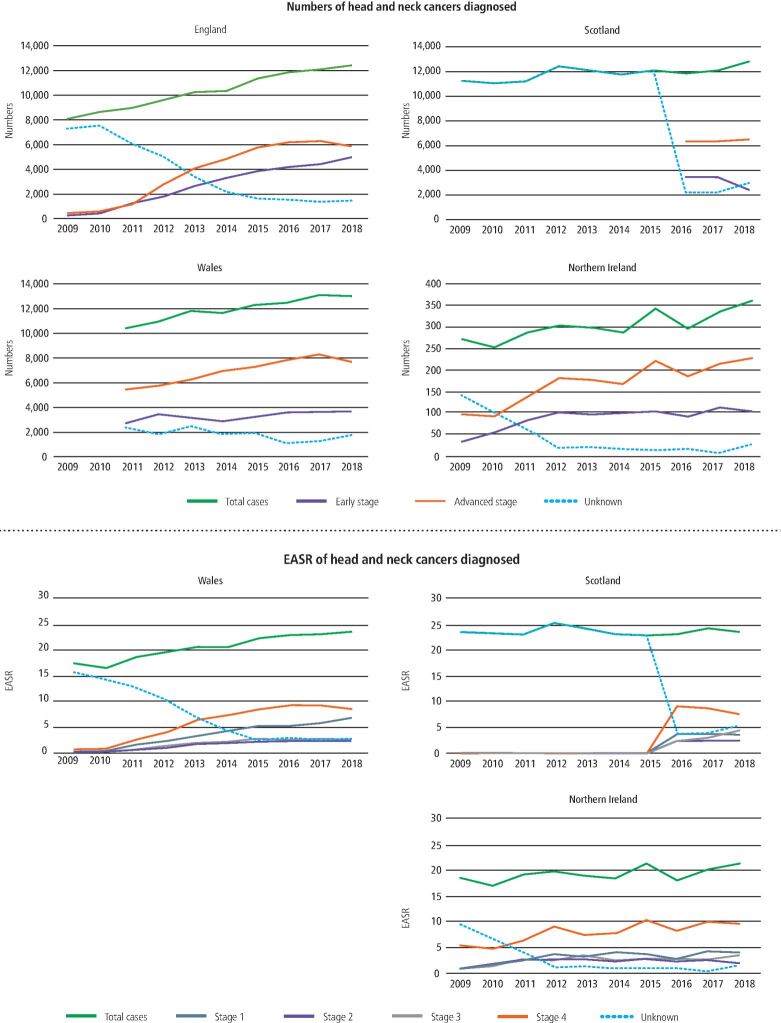
HNC incidence counts and rates by stage at diagnosis for each home nation for years 2009-2018

Across the four nations, proportions of HNCs diagnosed at an advanced-stage range from 50.5% in England, 52.6% in Scotland, 61.6% in Wales and 63.1% in Northern Ireland. When only analysing cases where the stage is recorded, these rates increase to 57.6% in England, 65.4% in Scotland, 68.9% in Wales and 66.9% in Northern Ireland (Fig. 3).

In terms of site groupings, oropharyngeal cancers have the highest proportion of advanced stage at diagnosis. Stage IV tumours account for the significant majority of new oropharyngeal disease in England. In Scotland, 61.3% of new oropharyngeal tumours are diagnosed at an advanced stage. This rises to 80.7% when only considering cases where the stage has been recorded (Fig. 3). Additional analysis utilising least square means shows oropharyngeal cancers to be the highest rate of cancer subsite across the UK and stage IV the most common stage at diagnosis (Table 2).

**Table 2:** Least square means tests for selected variables for EASR of new HNCs in England, Northern Ireland and Scotland 2009-2018 where stage is known

**Variable**		**EASR (95% CI)**	**Difference between means (95% CI)**
HNC subsite	Larynx	0.75 (0.58, 0.92)	1.00 (referent)
	Oropharyngeal	0.91 (0.74, 1.08)	0.16 (0.08, 0.41)
	Oral cavity	0.73 (0.55, 0.90)	-0.02 (-0.26, 0.22)
	Other sites of head and neck	0.76 (0.59, 0.94)	0.02 (-0.22, 0.26)
Stage at diagnosis	I	0.74 (0.59, 0.89)	1.00 (referent)
	II	0.44 (0.29, 0.59)	-0.31 (-0.52, -0.10)
	III	0.49 (0.34, 0.64)	-0.25 (-0.47, -0.04)
	IV	1.48 (0.33, 1.63)	0.74 (0.52, 0.95)

When considering cases in Scotland (Table 3), being aged 50 years or more (relative to under 50 years) at diagnosis was not associated with risk for presenting with advanced-stage disease (OR 1.10; 95% CI 0.86-1.42). Men tended more likely to be diagnosed at an advanced stage compared to women (OR 1.24; 95% CI 1.05-1.46). Although those from the most deprived areas (SIMD-5) were not found to have a statistically significant higher risks of advanced-stage HNC (OR 1.14; 95% CI 0.89-1.48), they did have a higher proportion of advanced-stage disease (68.9%) compared to those in the least deprived areas (63.7%). People diagnosed with oropharyngeal disease were statistically more likely to have advanced disease when compared to oral cavity disease (OR 3.14; 95% CI 2.54-3.89).

**Table 3:** HNC diagnoses by early/advanced stage in Scotland by age, sex and SIMD 2016-2018 where stage is known

**Demographic** **N = 1,016**		**Early stage**		**Advanced stage**		**Ratio advanced to early**	**Univariate model**		
		**34.57%**	**N = 1,923**	**65.43%**	**1.89**	**P Value**	**OR (to reference category)**	**(95% CI)**	
**Age at diagnosis**	<50 (ref)	104	36.62%	180	63.38%	1.73	-	1.00 (referent)	-
	50+	912	34.35%	1743	65.65%	1.91	0.222410	1.10	0.86-1.42
**Sex**	Female (ref)	324	38.03%	528	61.97%	1.63	-	1.00 (referent)	-
	Male	692	33.16%	1395	66.84%	2.02	0.005919	1.24	1.05-1.46
**Site grouping**	Oral cavity (ref)	392	42.98%	520	57.03%	1.33	-	1.00 (referent)	-
	Oropharynx	169	19.34%	705	80.66%	4.17	<0.0001	3.14	2.54-3.89
	Larynx	331	48.53%	351	51.47%	1.06	0.013853	0.80	0.65-0.98
	Other sites of head and neck	124	26.33%	347	73.67%	2.80	<0.0001	2.11	1.65-2.69
**SIMD**	5: least deprived (ref)	132	36.26%	232	63.74%	1.76	-	1.00 (referent)	-
	4	175	37.72%	289	62.28%	1.65	0.333869	0.94	0.71-1.25
	3	213	36.66%	368	63.34%	1.73	0.374067	0.96	0.73-1.26
	2	227	34.08%	439	65.92%	1.93	0.241447	1.10	0.84-1.44
	1	296	31.13%	595	68.87%	2.21	0.151167	1.14	0.89-1.48

## Discussion

These results show that the proportion of HNCs diagnosed with advanced disease has not improved across the UK in recent years, with 59% of people with HNC presenting with advanced-stage disease. In addition, data held by the national cancer registries show oropharyngeal cancer to be the most common subsite of HNC from 2009-2018 in the UK. There have been several large cohort studies incorporating stage at diagnosis into their investigations in recent decades. Our analyses show that from 2016-2018, 65% of HNCs with a known stage were diagnosed with advanced disease in Scotland, a higher proportion than that shown by the Scottish Audit of Head and Neck Cancer in 1999-2000, where 56% of HNCs were stage III or IV at diagnosis in the national population.^10^ The change has not been as pronounced in England, where the South West Audit of Head and Neck Cancer (1996-2003) had a 56-59% advanced-stage diagnostic rate,^11^ which is similar to that found here for England of 57% from 2009-2018. The UK-wide HN5000 study undertaken in 2011-2014 found 59.6% of all HNCs were diagnosed at an advanced stage, which again is similar to our findings for the UK, with 59% of HNCs diagnosed at advanced stage in the national registries.^12^ Care must be taken when interpreting these data, however, as there is a notable difference between the four nations, as described above. The data presented in this paper show that there has been no improvement in rates of advanced disease in the last 20 years, with current rates far higher currently in Scotland, Wales and Northern Ireland than in England. These rates across the UK fall short of the UK target of 75% of cancers being diagnosed at an early stage to be met by 2028.^23^

Cancer registries are population-level databases normally hosted within a public health system. They provide high-quality population coverage data on cancer incidence related to key demographics.^24^ With cancer registry data on HNCs becoming more complete over time, as demonstrated in this analysis, further opportunities emerge for better understanding the trends and burden of HNC in the UK. Appropriate understanding and use of cancer registry data has been demonstrated to be a very valuable asset for health providers and public bodies in reducing the burden of disease.^25^ Cancer stage at diagnosis is routinely recorded in registries for some major cancer sites, such as breast, colon and lung, but only recently has begun to be recorded for HNCs, as demonstrated here.

Although a rich and very useful source of population level data, cancer registry databases do have limitations. Registries rely on accurate data being recorded and entered from the clinical locations and while there are high levels of quality and completeness in cancer registries overall, some fields, particularly stage, have been incomplete.^26^^,^^27^^,^^28^ Analysis of cancer registry data for other types of cancer has suggested that those cases with missing stages within cancer registries may be more likely to be from people diagnosed with later-stage disease, meaning that the proportion of advanced disease may in fact be underreported. This has not been shown for HNCs to-date, however, although it may suggest some underestimation of our study findings.^29^

Advanced stage at diagnosis of an HNC is defined as stage III or IV according to the eighth edition of TNM.^21^ There was one significant change in the transition from the seventh edition to the current volume, involving P16 positive tumours of the oropharynx. This change, a downstaging for certain P16-positive oropharyngeal tumours given the more favourable prognosis of these compared to P16-negative tumours, was able to be adopted by all registries from January 2018 and will inevitably cause some heterogeneity in data-recording processes. This change could have resulted in the disease being classified as early rather than advanced stage, meaning that any reduction in the rate of advanced stage at diagnosis observed in 2018 may be, in part, a disease classification phenomenon as opposed to a true reduction in severity of disease presentation.

The importance of the stage at diagnosis as a prognostic factor cannot be understated. Stage at diagnosis is one of the key prognostic indicators of mortality in people with HNC,^5^ with an increase in morbidity and reduction in post-treatment quality of life also observed.^30^ There are many factors that influence the stage at diagnosis of HNC, including patient-related factors, tumour-related factors, human papillomavirus infection and socioeconomic factors.^6^^,^^31^^,^^32^^,^^33^^,^^34^ While not found to be statistically different, the result that those in the most socioeconomically deprived areas of Scotland (SIMD-1) have a higher proportion of advanced-stage disease when compared to those in the least deprived areas (SIMD-5) suggests that there are wider socioeconomic environmental factors that potentially influence the stage at diagnosis of HNC. A key area for further research is that of health-system factors in the stage of diagnosis. It is widely reported that delays in diagnosis of HNC can have a significant impact on the tumour-related and patient-related outcomes, with delay coming from both the patient interval and professional interval.^35^ In order to improve rates of early diagnosis, more understanding is required as to how these delays can be addressed, something that a major World Health Organisation and International Agency for Research on Cancer-led initiative, the Head and Neck Cancer in South America and Europe (HEADSpAcE) study, is currently investigating.^36^

A key reason for the importance of this study is to understand the pre-pandemic situation with regards to stages at diagnosis of HNC to accurately ascertain the effect that the COVID-19 pandemic has had on the HNC system in the UK. It is possible that rates of advanced disease will rise given the well-documented issues of limited access to health services and reported lower rates of diagnosis generally; however, emerging research from European countries has not demonstrated this.^37^ While HNC services have been able to continue diagnosing and treating patients throughout the COVID-19 pandemic,^38^ there is concern that the pandemic may have led to more patients presenting with advanced disease, or not at all, to health services. It will be important to monitor the impact of the COVID-19 pandemic on the incidence of advanced-stage HNC and we have shown there is improving data available via the UK cancer registries which can be used to monitor this and inform service recovery.

For dentists, oral cancer is a key topic for ongoing continuing professional development.^39^ Most patients first present to primary care in the first instance with a HNC but there is more that can potentially be done in raising awareness across health professionals^40^ and also in the general public^41^ in order to reduce rates of advanced disease. Dentists can play a very important role in the early detection of HNC through opportunistic screening and more frequent recall intervals for patients who may be at higher risk of developing HNC despite low-detection rates in primary care.^14^ An updated recent Cochrane review reiterated the the important role that frontline oral health teams play in detecting oral malignancies.^42^ However, for this to be the case, clear pathways and guidelines have to be available for referral of suspicious signs and symptoms to secondary care settings, as advised in the recent *Delivering better oral health* guidelines.^43^ National guidelines also exist to facilitate this pathway, such as the National Institute for Health and Care Excellence Guideline 12^44^ and the Scottish Cancer Referral Guidelines.^45^

## Conclusions

Prior to the COVID-19 pandemic, based on available population data, diagnoses of HNC at an advanced stage comprised the majority of HNCs across the UK, representing the major challenge for the cancer healthcare system, including the need for better clarification of the role of the dental team and their links with/referral pathways to HNC services. Understanding the reasons behind the high levels of advanced HNC at presentation is vital in order to reduce the substantial impact of this disease and the poor survival rates experienced in the UK and internationally.

The future trends of advanced-stage HNC as we emerge from the COVID-19 are not yet known but these data demonstrate a pre-pandemic baseline level which highlights this as a significant public health challenge.

## Supplementary Information

Supplementary Table 1 (PDF 190KB)
