# Supplementary material for: Head and neck cancer in the UK: what was the stage before COVID-19? UK cancer registries analysis (2011-2018)
Source: Br Dent J. 2022 Nov 11;233(9):787–93. doi: 10.1038/s41415-022-5151-4 (PMC9650177; doi:10.1038/s41415-022-5151-4)
Supplement: Supplementary file 1 — Supplementary Table 1 (PDF 190KB) [file 41415_2022_5151_MOESM1_ESM.pdf]

Supplementary Table 1 Counts and ASR for new HNCs by stage at diagnosis by variables available

		Numbers of cases by stage					***ASR by stage***				
		I	***II***	***III***	***IV***	***Unknown***	***I***	***II***	***III***	***IV***	***Unknown***
England											
Subsite all years available	Oral cavity cancer (OCC)	4511	1904	1200	5782	6509	N/A	N/A	N/A	N/A	N/A
	Oropharyngeal cancer (OPC)	1298	1554	2914	13690	7660	N/A	N/A	N/A	N/A	N/A
	Larynx	4144	2068	2100	3173	6281	N/A	N/A	N/A	N/A	N/A
	Other	9313	2845	3181	7150	24515	N/A	N/A	N/A	N/A	N/A
	All HNC	19266	8371	9395	29795	45010	N/A	N/A	N/A	N/A	N/A
Year	2008	193	103	114	272	6979	0.4	0.2	0.2	0.5	10.7
	2009	211	116	107	331	7368	0.5	0.3	0.2	0.7	12.5
	2010	308	162	184	450	7614	0.6	0.3	0.4	1.0	11.2
	2011	841	402	395	1267	6131	1.7	0.9	0.8	2.7	10.4
	2012	1233	585	722	2068	5065	2.5	1.2	1.5	4.3	9.2
	2013	1784	906	1007	3160	3435	3.5	1.8	2.1	6.4	6.2
	2014	2277	1034	1159	3736	2212	4.5	2.1	2.3	7.5	3.9
	2015	2732	1195	1432	4368	1682	5.3	2.4	2.8	8.6	2.9
	2016	2853	1247	1417	4796	1574	5.4	2.4	2.7	9.3	2.7
	2017	3133	1298	1463	4834	1425	5.9	2.5	2.8	9.3	2.4
	2018	3701	1323	1395	4513	1525	6.9	2.5	2.7	8.6	2.5
Northern Ireland											
Subsite all years available	OCC	181	79	58	327	156	N/A	N/A	N/A	N/A	N/A

	OPC	45	69	155	502	78	N/A	N/A	N/A	N/A	N/A
	Larynx	241	161	145	179	81	N/A	N/A	N/A	N/A	N/A
	Other	46	70	72	268	125	N/A	N/A	N/A	N/A	N/A
	All HNC	513	379	430	1276	440	N/A	N/A	N/A	N/A	N/A
Year	2009	16	17	15	82	141	1.2	1.2	1.0	5.6	9.6
	2010	29	27	23	71	103	1.9	1.8	1.5	5.0	6.9
	2011	41	44	43	96	64	2.8	2.9	2.8	6.4	4.3
	2012	59	43	40	142	20	3.8	2.8	2.6	9.2	1.3
	2013	52	46	57	120	23	3.3	2.9	3.6	7.7	1.5
	2014	64	39	43	125	17	4.1	2.5	2.7	8.0	1.2
	2015	61	44	51	171	16	3.8	2.8	3.1	10.6	1.1
	2016	51	41	50	136	18	3.1	2.5	3.0	8.4	1.1
	2017	70	43	46	168	9	4.3	2.6	2.8	10.1	0.6
	2018	70	35	62	165	29	4.2	2.0	3.6	9.8	1.7
Scotland											
Subsite all years available	OCC	271	121	110	410	88	1.7	0.8	0.7	2.6	0.6
	OPC	83	86	182	523	276	0.5	0.5	1.1	3.3	1.7
	Larynx	203	128	169	182	161	1.3	0.8	1.1	1.2	1.1
	Other	57	67	76	271	195	0.4	0.4	0.5	1.8	1.3
	All HNC	614	402	537	1386	720	3.9	2.6	3.4	8.9	4.6
Year	2016	207	132	137	492	211	4.1	2.5	2.7	9.5	4.1
	2017	205	137	167	478	216	3.9	2.6	3.2	9.2	4.2
	2018	202	133	233	416	293	3.8	2.5	4.3	7.9	5.6

Sex	Men	403	289	389	1006	496	5.4	3.8	5.1	13.3	6.7
	Women	211	113	148	380	224	2.5	1.3	1.7	4.4	2.6
SIMD Category	SIMD1	155	114	165	430	226	5.6	4.1	5.9	15.4	8.2
	SIMD2	125	102	120	319	152	4.0	3.3	3.9	10.4	5.1
	SIMD3	145	68	99	269	148	4.4	2.1	3.0	8.1	4.5
	SIMD4	107	68	80	209	114	3.2	2.0	2.4	6.4	3.4
	SIMD5	82	50	73	159	80	2.6	1.6	2.3	5.2	2.5
Age	0 to 4	0	0	0	0	1	0.0	0.0	0.0	0.0	0.1
	5 to 9	0	0	0	1	0	0.0	0.0	0.0	0.1	0.0
	10 to 14	0	0	1	0	1	0.0	0.0	0.1	0.0	0.1
	15 to 19	0	1	0	1	0	0.0	0.1	0.0	0.1	0.0
	20 to 24	4	0	1	2	2	0.4	0.0	0.1	0.2	0.2
	25 to 29	2	4	1	2	0	0.2	0.4	0.1	0.2	0.0
	30 to 34	5	1	2	6	8	0.5	0.1	0.2	0.6	0.7
	35 to 39	10	2	4	23	3	1.0	0.2	0.4	2.3	0.3
	40 to 44	13	8	17	35	16	1.3	0.8	1.7	3.6	1.6
	45 to 49	31	23	27	57	43	2.7	2.0	2.3	4.9	3.7
	50 to 54	52	37	64	136	60	4.3	3.0	5.3	11.2	4.9
	55 to 59	75	54	79	193	78	6.6	4.8	7.0	17.0	6.9
	60 to 64	102	58	82	228	125	10.3	5.9	8.3	23.1	12.7
	65 to 69	95	73	97	232	115	10.3	7.9	10.5	25.1	12.5
	70 to 74	78	57	69	180	98	10.1	7.4	9.0	23.4	12.7
	75 to 79	78	43	51	136	64	13.8	7.6	9.0	24.0	11.3

	80 to 84	45	26	23	90	64	10.9	6.3	5.5	21.7	15.4
	85 to 89	19	9	14	42	23	7.9	3.8	5.8	17.5	9.6
	Over 90	5	6	5	22	19	4.0	4.8	4.0	17.6	15.2
Wales											
Subsite all years available	OCC + OPC	424	280	153	1210	410	N/A	N/A	N/A	N/A	N/A
	Larynx	329	185	244	296	114	N/A	N/A	N/A	N/A	N/A
Year	All HNC	858	623	601	2567	821	N/A	N/A	N/A	N/A	N/A
	2011	98	54	69	242	129	N/A	N/A	N/A	N/A	N/A
	2012	109	85	48	280	102	N/A	N/A	N/A	N/A	N/A
	2013	85	90	46	311	142	N/A	N/A	N/A	N/A	N/A
	2014	85	78	74	324	105	N/A	N/A	N/A	N/A	N/A
	2015	97	85	79	334	110	N/A	N/A	N/A	N/A	N/A
	2016	124	79	85	363	63	N/A	N/A	N/A	N/A	N/A
	2017	133	71	100	374	73	N/A	N/A	N/A	N/A	N/A
Sex	2018	127	81	100	339	97	N/A	N/A	N/A	N/A	N/A
	Men	573	418	447	1895	550	N/A	N/A	N/A	N/A	N/A
	Women	285	205	154	672	271	N/A	N/A	N/A	N/A	N/A
